# Vodka eyeballing: a potential cause of ocular injuries

**DOI:** 10.5249/jivr.v6i2.540

**Published:** 2014-07

**Authors:** Anand N. Bosmia, Christoph J. Griessenauer, Richard Shane Tubbs

**Affiliations:** ^*a*^Children’s of Alabama, Pediatric Neurosurgery, Birmingham, Alabama, USA.; ^*b*^Division of Neurosurgery, Department of Surgery, The University of Alabama at Birmingham, Birmingham, Alabama, USA.

Physicians may encounter patients with acute injuries or chronic problems secondary to performing dares promoted on the Internet through websites such as YouTube and Facebook. Such websites provide a window into trends that illuminate transgressive behavior among teenagers and young adults. ^1^ In 2010, reports in the media emerged concerning a practice known as “vodka eyeballing”, which involves a person pouring shots of vodka directly into the eye. Vodka eyeballing may have originated in the United States as a trick performed by waitresses for tips at resorts. A 2000 film called Kevin and Perry go large has been blamed for encouraging vodka eyeballing by featuring an actor who performs the stunt.^2^

20-proof alcohol is used to delaminate the epithelial basement membrane of the cornea in laser subepithelial keratectomy.^3^ Vodka contains more ethanol by volume than this solution, and has corrosive effects on the eye. Physicians report that vodka eyeballing can cause corneal abrasions and scarring,^3,4^ promote angiogenesis in the eye and thereby cause loss of vision,^5,6^ and increase the risk for ocular infections.^4^

Participants claim that alcohol enters the bloodstream through veins in the rear of the eye and thereby causes speedier inebriation than drinking does. The American Academy of Ophthalmology states that a person does not get a “quick high” because the amount of alcohol absorbed by the conjunctiva and cornea is too small to have such an effect.^4^ Furthermore, participants already may be drunk at the time of the act and thus mistakenly think that vodka eyeballing intoxicates them more quickly.^3^

College students are at risk for engaging in vodka eyeballing. Participation in dares such as vodka eyeballing is partly driven by a desire to demonstrate one’s bravado to his or her peers in the milieu of the university drinking culture of the United States and the United Kingdom.^2^ This mentality, coupled with impaired judgment secondary to intoxication with alcohol, can inspire participants to make the dare even more dangerous. Participants may use beverages with higher alcohol content by volume or take shots in both eyes at the same time. These individuals may present to general practitioners or emergency physicians with diminished vision, tenderness of the eye, or weeping from the eye,^2^ and should be referred to an ophthalmologist.

In conclusion, general practitioners, emergency physicians, and ophthalmologists may encounter patients who present with ocular problems secondary to vodka eyeballing. This practice has become more prevalent among college students per reports in the media that emerged in 2010.
